# Treatment of multiple adjacent RT 1 gingival recessions with the modified coronally advanced tunnel (MCAT) technique and a collagen matrix or palatal connective tissue graft: 9-year results of a split-mouth randomized clinical trial

**DOI:** 10.1007/s00784-022-04674-9

**Published:** 2022-08-22

**Authors:** B. Molnár, S. Aroca, A. Dobos, K. Orbán, J. Szabó, P. Windisch, A. Stähli, A. Sculean

**Affiliations:** 1 Department of Periodontology, Semmelweis University, Budapest, Hungary; 2 Department of Periodontology, School of Dental Medicine, University of Bern, Bern, Switzerland

**Keywords:** Modified coronally advanced tunnel, Multiple adjacent gingival recessions, Subepithelial connective tissue graft, Collagen matrix

## Abstract

**Objectives:**

To evaluate t he long-term outcomes following treatment of RT 1 multiple adjacent gingival recessions (MAGR) using the modified coronally advanced tunnel (MCAT) with either a collagen matrix CM or a connective tissue graft (CTG).

**Material and methods:**

Sixteen of the original 22 subjects included in a randomized, controlled split-mouth clinical trial were available for the 9-year follow-up (114 sites). Recessions were randomly treated by means of MCAT + CM (test) or MCAT + CTG (control). Complete root coverage (CRC), mean root coverage (MRC), gingival recession depth (GRD), probing pocket depth (PD), keratinized tissue width (KTW), and thickness (KGT) were compared with baseline values and with the 12-month results.

**Results:**

After 9 years, CRC was observed in 2 patients, one in each group. At 9 years, MRC was 23.0 ± 44.5% in the test and 39.7 ± 35.1% in the control group (*p* = 0.179). The MRC reduction compared to 12 months was − 50.1 ± 47.0% and − 48.3 ± 37.7%, respectively. The upper jaw obtained 31.92 ± 43.0% of MRC for the test and 51.1 ± 27.8% for the control group (*p* = 0.111) compared to the lower jaw with 8.3 ± 46.9% and 20.7 ± 40.3%. KTW and KGT increased for both CM and CTG together from 2.0 ± 0.7 to 3.1 ± 1.0 mm (< 0.0001). There were no statistically significant changes in PD.

**Conclusion:**

The present results indicate that (a) treatment of MAGR using MCAT in conjunction with either CM or CTG is likely to show a relapse over a period of 9 years, and (b) the outcomes obtained in maxillary areas seem to be more stable compared to the mandibular ones.

**Clinical relevance:**

The mean root coverage at 12 months could not be fully maintained over 9 years. On a long-term basis, the results seem to be less stable in the mandible as compared to maxillary areas.

## Introduction

Gingival recession (GR) is defined as the apical shift of the gingival margin with respect to the cemento-enamel junction (CEJ), associated with attachment loss and exposure of the root surface to the oral environment [1, 2]. GR is commonly observed, especially among young and middle-aged adults [3]. Besides aesthetic complaints, GR may also cause root hypersensitivity, risk for development of caries or non-carious cervical lesions, and difficulties to achieve optimal plaque control [4].

While most of the existing literature reports on the treatment of single gingival recessions [5, 6], frequently, root exposures affect multiple adjacent teeth and are considered a generalized condition [7, 8]. The treatment of multiple adjacent gingival recessions (MAGR) poses a challenge for the clinician while data is still scarce on these procedures [9]. In the last two decades, the modified coronally advanced flap (MCAF) has become one of the most popular techniques for the treatment of MAGR [10]. Another surgical approach which has provided successful outcomes in the treatment of MAGR is the modified coronally advanced tunnel (MCAT) consisting of a preparation without raising a mucosal or mucoperiosteal flap and keeping the papillae intact. Several studies have recently evaluated the treatment of single and multiple recessions with MCAT demonstrating comparable improvements to those following the use of MCAF [11–14].

MCAT with the absence of releasing incisions delivers aesthetic outcomes; other benefits are favorable wound healing, minimal postoperative morbidity, and optimal blood supply and graft nutrition [15].

Palatal connective tissue graft (CTG) is still the gold standard among the soft tissue grafts used for various soft tissue augmentation procedures, although limitations in the size, shape, and thickness homogeneity may be present [7]. However, CTG harvesting may be associated with prolonged surgical time, increased patient morbidity, and the possibility of postoperative complications. To overcome these inconveniences, there has been a strong demand to find an alternative soft tissue grafting material [16]. The use of a porcine xenogeneic collagen matrix (CM; Mucograft, Geistlich, Wolhusen, Switzerland) in recession coverage was first evaluated in a histological study in minipigs. CM can serve as a scaffold for cells to enhance blood clot stability and conduce the ingrowth of blood vessels. Allergic reactions and material exfoliations were not reported during the application of this CM for recession coverage [17]. Clinically, a case report [18] as well as randomized controlled clinical studies compared the treatment of Miller class I and II single [19] and later multiple recessions [15, 20]. Several articles have compared sites treated by gingival augmentation to untreated sites in long-term studies with 18–35-year follow-up. The long-term observations support the importance of attached gingiva in preventing recession development due to prolonged mechanical trauma, bacterial inflammation, and iatrogenic factors during aging [1, 21]. A study with a long-term follow-up showed that sites treated with autologous soft tissue graft transplantation showed coronal displacement of the gingival margin with recession reduction, whereas recessions at contralateral untreated sites increased, or new recessions were developed during an 18- to 35-year follow-up [21].

Despite the fact that CM was proven to be a realistic alternative to CTG on the short term (i.e., after a period of 12 months), long-term results are still missing in the literature.

Therefore, the aim of the present study was to evaluate the long-term outcomes (i.e., after a period of 9 years) following treatment of class 1 (previously Miller class I and II) MAGR by means of the MCAT and either CM or CTG.

## Materials and methods

The CONSORT statement for improving the quality of reports of parallel RCT (http://www.consort-statement.org/) was followed in the preparation of this study.

### Study design and patient demographics

This is a 9-year follow-up of a previously published randomized split-mouth study [15], involving twenty-two patients with a total of 156 sites of class 1 (previously Miller I and II) MAGR [22, 23]. The original study was conducted between July 2010 and November 2011 at the Department of Periodontology of the Semmelweis University, Budapest, Hungary, in accordance with the Helsinki Declaration of 1975, as revised in 2013. The study protocol was approved by the ethical committee of the Semmelweis University (protocol: 5242–0/2010-101SEKU; 365/PI/10). The detailed protocol of the study along with the outcomes obtained at 1 year has been published before [15]. Thus, in the following, only a summary of the study design and patient demographics is presented.

To detect a true difference for the primary outcome of 20% assuming a power of 80%, the sample size calculation requested a minimal sample size of 18 patients. A total of 22 patients were finally included.

### Inclusion criteria

The inclusion criteria are the following: 18 years old or older, systemically healthy subjects with at least 3 adjacent Miller I and II recessions on both sides. Full-Mouth Plaque Score (FMPS) had to be under 25%.

### Exclusion criteria

The exclusion criteria are the following: pregnancy or lactation, tobacco smoking, uncontrolled medical conditions, medications that can affect gingival conditions, infectious diseases, non-cooperative patients, failure to sign informed consent.

### Surgical approach

All the 22 patients underwent full-mouth supragingival scaling and polishing; then, individualized oral hygiene instructions were given preoperatively. The modified coronally advanced tunnel technique (MCAT) was applied in all cases in conjunction with either CM or CTG in a randomized split-mouth design. The random allocation of groups was generated using a computer program. Thus, every patient had one side of the jaw treated by means of MCAT technique with a bioresorbable collagen matrix (Mucograft®, Geistlich, Wolhusen, Switzerland) as a test. The other side of the jaw was treated with CTG, which was harvested from the palate and this site was considered control. Both surgeries were performed by the same experienced surgeon (SA) in one session. The surgical technique was described in detail in a previous article [18]. In brief, resin bonding at the contact points of the adjacent teeth was performed immediately before the surgery for suspended suturing. Under local anesthesia, the exposed root surfaces were gently planed by hand instruments (Gracey Curettes, Hu-Friedy). The preparation of MCAT started with an intrasulcular incision around involved teeth using microsurgical tunneling knives (Stoma). Mucoperiosteal flaps were elevated first as an envelope flap subsequently interconnected in a tunnel preparation. Flap preparation was extended beyond the mucogingival junction in split thickness and lastly, interdental papillae were gently undermined to allow tension-free, passive mobilization to the coronal aspect. After tunnel preparation, the grafting procedure was carried out according to the randomization code. The collagen matrix was trimmed and adapted to the required length and size. A CTG was harvested from the palate by the single incision technique [24] or a modified distal wedge procedure [25]. To close the donor site, either cross-mattress sutures or modified mattress sutures were placed. The insertion of both grafts to the subperiosteal tunnel was started in the widest recession using horizontal mattress sutures at mesial and distal aspect of the grafts. Finally, suspended sutures were placed above the approximal composite stops, and the tunneled flap was positioned 1 mm coronally to the cemento-enamel junction (CEJ).

### Post-surgical treatment

Patients were prescribed analgesics for 3 days (3 × 50 mg diclofenac, Cataflam, Novartis) and antibiotics (3 × 625 mg amoxicillin and clavulanic acid, Augmentin, GlaxoSmithKline) for 7 days for infection prophylaxis. In addition, patients were advised to rinse with a 0.2% chlorhexidine solution (2 × per day for 3 weeks) and to discontinue tooth brushing at the surgical sites until the suture removal (2 weeks). The composite stops at the contact points were removed after 3 weeks. Recall appointments were scheduled every 3 months in the first 1 year and every 6 months thereafter.

### Measurements

Clinical assessments were performed at baseline, at 1 month, 3 months, 6 months, 12 months and 9 years after the surgery by the same masked investigator (BM). Assessed parameters were as follows: recession depth (RD), recession width [11], keratinized gingiva width (KGW) in the mid-buccal aspect, papilla-contact point distance (PCD), papilla width (PW), probing depth on 3 surfaces (PD). Keratinized tissue thickness (KT) was only measured at baseline, 6 months, 12 months, and 9 years via sterile K-files following local anesthesia at 3-mm apical distance from the gingival margin. A UNC 15 type periodontal probe was used for measurements (Hu-Friedy, Chicago, IL, USA) The examiner was calibrated as discussed in original article. During surgery, the length of the procedure was measured in minutes.

Statistical analysis was performed using commercially available software (Instats 2000, version 3.05, GraphPad Prism 9.0.0. Software Inc., San Diego, CA, USA). A patient-level analysis was performed for each parameter. Therefore, mean values and standard deviations [26] for the clinical variables were calculated for each patient per treatment. The primary outcome variable was complete root coverage (CRC); secondary outcomes were MRC, amount of KTT, KTW, GRD, PW, and PD, respectively.

### Statistical analysis

For each clinical parameter, a patient-level analysis was performed; i.e., mean values and standard deviations were calculated for each outcome and patient, respectively. Due to the non-parametric distribution of the data, between-group comparisons including Bonferroni corrections were conducted using the Mann–Whitney *U* test for independent unpaired variables, the Wilcoxon signed rank test for paired, and the Friedman test for dependent variables. Significance was set at *p* < 0.05.

## Results

### Original study with 12-month results

Detailed demographics and description of the original study sample were reported in the original publication [15].

Data in the original article represented split mouth data of 22 patients with 156 recessions (78 treated with CTG and 78 with CM). Mean root coverage (MRC) was 71% on test and 90% on control sites at 12 months. Complete root coverage (CRC) of all treated teeth was achieved in 5 sides out of 22 in the test and in 13 sides out of 22 in the control, respectively. Three patients presented with CRC for all treated recessions. Mean KGW had increased from 2.1 ± 0.9 to 2.4 ± 0.7 mm on test sites and from 2.0 ± 0.7 to 2.7 ± 0.8 mm on the control sites. KGT had increased from 0.8 ± 0.3 to 0.95 ± 0.4 mm (test) and from 0.8 ± 0.4 to 1.3 ± 0.5 mm (control). PD had not changed.

### Patients’ characteristics at 9-year follow-up

Sixteen out of 22 (72%) individuals were available for a 9-year recall with a total of 114 treated recessions (72 in the maxilla and 42 in the mandible). None of the patients had received restorations or lost any of the investigated teeth during the 9 years of follow-up (Table 1).

**Table 1:** Patient demographics

	Baseline–9 years	
	Baseline	9 years
Number of patients	22	16
Number of total recessions	156	114
Number of maxillary recessions	94	72
Number of mandibular recessions	62	42

### Clinical outcome after 9 years

#### MRC

Results are compiled in Table 2. Compared to 12 months, MRC decreased from 73.2 ± 21.0 to 23.0 ± 44.5% in the test and from 88.0 ± 20.9 to 39.7 ± 35.1% in the control among the 16 subjects who attended the 9-year follow-up. Differences in MRC between the groups were significant after 12 months (*p* = 0.021) but diminished after 9 years (*p* = 0.179). In the mandible, MRC decreased from 79.7 ± 18.9% at 12 months to 8.3 ± 46.9% at 9 years in the test group, and from 95.8 ± 6.6 to 20.7 ± 40.3% in the control group, respectively. In the maxilla, MRC decreased from 69.3 ± 22.2 to 31.9 ± 43.0% (test) and 83.4 ± 25.3 to 51.1 ± 27.8% (control). After 9 years, CRC was maintained on 32 teeth out of 114 treated recessions. There was one side in each group that reached CRC for all treated teeth of the quadrant.

**Table 2:** Mean and complete root coverage

	Baseline–12 months			Baseline–9 years		
	Test group (MCAT + CM)	Control group (MCAT + CTG)	*p* value	Test group (MCAT + CM)	Control group (MCAT + CTG)	*p* value
Mean root coverage (MRC) %	73.25 ± 21.05	88.07 ± 20.90	0.021	23.07 ± 44.56	39.73 ± 35.17	0.179
MRC maxilla %	69.38 ± 22.23	83.44 ± 25.31	0.109	31.92 ± 43.06	51.11 ± 27.80	0.111
MRC mandible %	79.71 ± 18.98	95.77 ± 6.05	0.187	8.83 ± 46.92	20.75 ± 40.36	> 0.99
Teeth with complete root coverage (CRC)	31	45		14	18	
Patients with CRC for all teeth on one side	4	9		1	1	

#### Keratinized tissue gain compared to baseline

In terms of keratinized tissue volume gain (Table 3), KTW increased by 0.9 ± 1.1 mm for test and by 0.8 ± 1.0 mm for the control group (*p* = 0.7197) whereas KTT gained by 0.6 ± 0.3 mm in the test and 0.7 ± 0.3 mm in the control group (*p* = 0.8403). When splitting up the results for mandible and maxilla, in the mandible, the increase was 1.7 ± 0.26 mm for the test group and 1.3 ± 1.3 mm for the control group (*p* = 0.625) and in the maxilla 0.9 ± 1.3 mm and 0.47 ± 0.6 mm (*p* = 0.3047), respectively. Regarding KTT, in the mandible, the increase was 0.7 ± 0.3 mm and 0.7 ± 0.3 mm (*p* = 0.6562) for test and control. In the maxilla, KTT increased by 0.58 ± 0.4 mm for the test and 0.6 ± 0.28 mm for the control group (*p* > 0.9).

**Table 3:** Mucogingival parameters

	Test group (MCAT + CM)	Control group (MCAT + CTG)	*p* value *t* vs *c*
Recession depth (RD)			
Baseline (*t*_1_)	1.81 ± 0.63	1.78 ± 0.54	0.900
12 months (*t*_2_)	0.50 ± 0.40	0.21 ± 0.30	0.035
9 years (*t*_3_)	1.28 ± 0.68	1.06 ± 0.65	0.635
* p* value *t*_1_ vs *t*_2_	< 0.0001	< 0.0001	
* p* value *t*_2_ vs *t*_3_	< 0.0001	< 0.0001	
* p* value *t*_1_ vs *t*_3_	ns	ns	
Keratinized tissue thickness (KTT)			
Baseline (*t*_1_)	0.833 ± 0.26	0.86 ± 0.29	0.515
12 months (*t*_2_)	0.97 ± 0.31	1.32 ± 0.40	0.0002
9 years (*t*_3_)	1.49 ± 0.32	1.57 ± 0.35	0.710
* p* value *t*_1_ vs *t*_2_	ns	< 0.0001	
* p* value *t*_2_ vs *t*_3_	< 0.0001	ns	
* p* value *t*_1_ vs *t*_3_	< 0.0001	< 0.0001	
Keratinized tissue width (KTW)			
Baseline (*t*_1_)	2.00 ± 0.90	2.03 ± 0.65	0.845
12 months (*t*_2_)	2.32 ± 0.72	2.78 ± 0.82	0.074
9 years (*t*_3_)	2.97 ± 0.95	3.28 ± 1.14	0.336
* p* value *t*_1_ vs *t*_2_	ns	ns	
* p* value *t*_2_ vs *t*_3_	ns	ns	
* p* value *t*_1_ vs *t*_3_	0.002	0.0006	
Papilla width (PW)			
Baseline (*t*_1_)	3.54 ± 0.80	3.62 ± 0.86	0.968
12 months (*t*_2_)	4.65 ± 1.06	4.92 ± 1.16	0.189
9 years (*t*_3_)	4.67 ± 0.66	4.54 ± 0.53	0.425
* p* value *t*_1_ vs *t*_2_	0.0003	0.0002	
* p* value *t*_2_ vs *t*_3_	Ns	Ns	
* p* value *t*_1_ vs *t*_3_	0.0003	0.0002	

#### Keratinized tissue gain compared to 12 months

The average gain in KTW at 9 years was 0.6 ± 0.9 mm and 0.4 ± 0.6 mm for CM and CTG (*p* = 0.7168). KTT revealed an increase following both procedures of 0.5 ± 0.4 mm in the test and 0.2 ± 0.3 mm in the control with a significant difference between the groups (*p* = 0.0259).

In the mandible, KTW increased by 0.3 ± 0.7 mm in the test and 0.5 ± 0.6 mm in the control (*p* = 0.375). The corresponding values for the maxilla were 0.8 ± 1.0 mm for the test and 0.4 ± 0.6 mm for the control (*p* = 0.2969). In the mandible, KTT increased by 0.5 ± 0.3 mm in the test and 0.28 ± 0.3 mm in the control group (*p* = 0.0625). In the maxilla, for KTT, the increase from 12 months to 9 years were as follows: 0.47 ± 0.4 mm in the test, 0.17 ± 0.37 mm in the control group (*p* = 0.1992).

#### Measurements of papilla-contact point distance (PCD) and papilla width (PW)

PCD presented no differences between test and control for any timepoint whereas PW demonstrated a significant increase at 12 months compared to baseline for both groups. This difference was maintained over the 9 years (Table 3).

#### Pocket depth (PD)

No differences of pocket depths were found between the groups and timepoints.

**Fig. 1:**
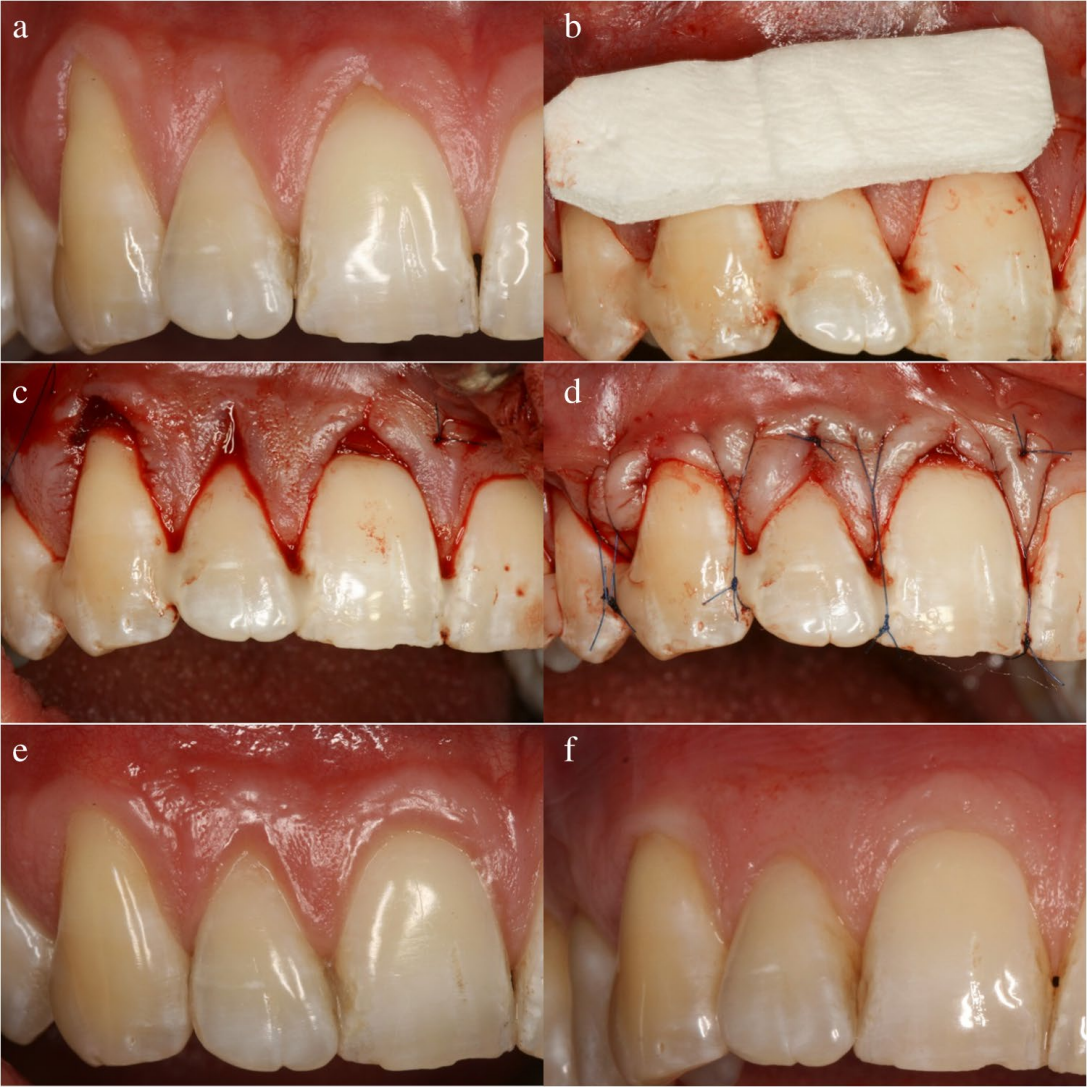
Case Nr. 19. Test side treatment and follow-up — **a** baseline, **b** CM adaptation, **c** CM in tunnel, **d** suspended suturing, **e** 1 year, **f** 9 years/CM, mucograft collagen matrix

**Fig. 2:**
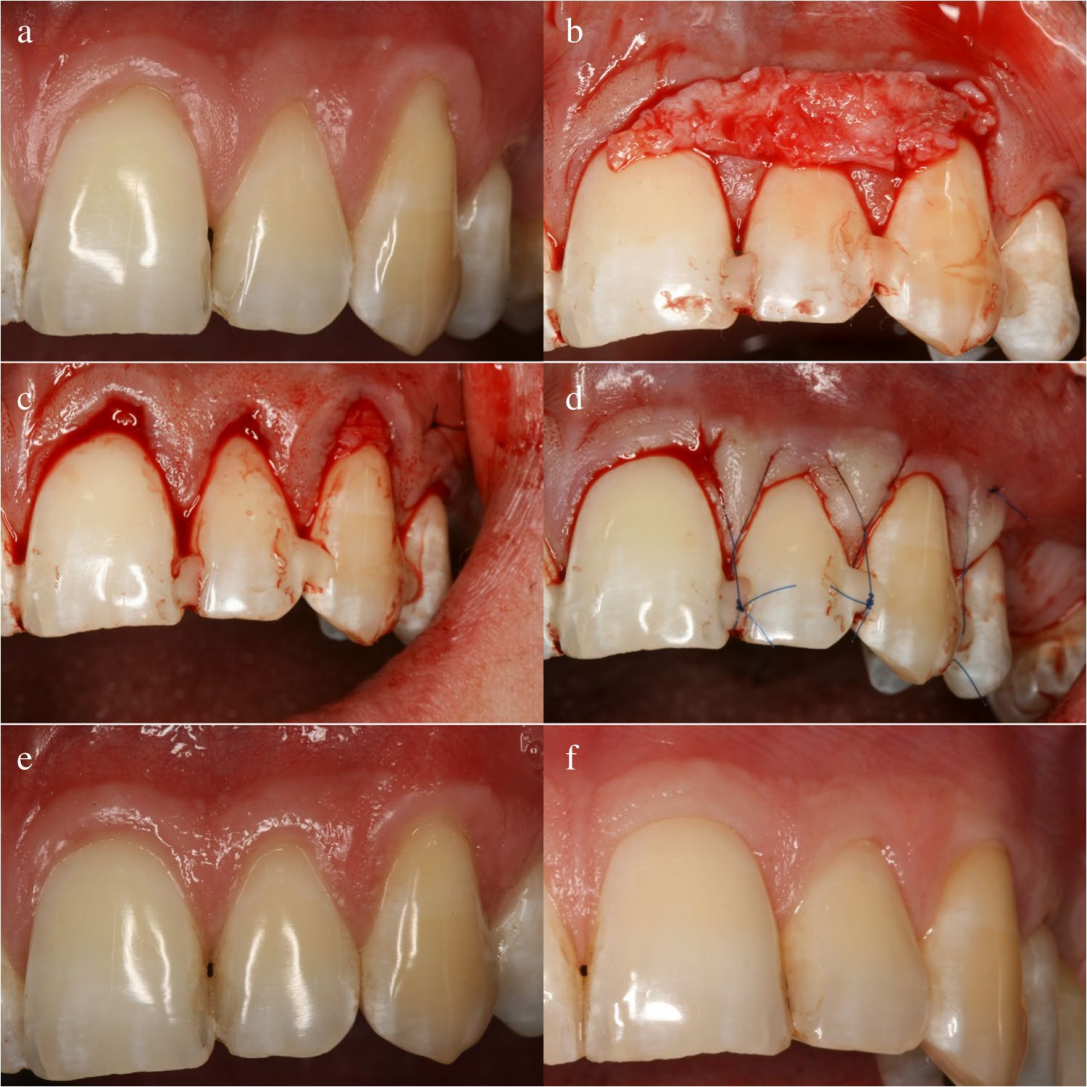
Case Nr. 19. Control side treatment and follow-up — **a** baseline, **b** CTG adaptation, **c** CTG in tunnel, **d** suspended suturing, **e** 1 year, **f** 9 years/CTG, connective tissue graft

Clinical procedures and outcomes are represented in Fig. 1 and Fig. 2.

## Discussion

The present study has evaluated the long-term outcomes following treatment of class 1 MAGR using the MCAT in conjunction with either CM or CTG. The results revealed that both graft materials may lead to positive aesthetic outcomes, which can be maintained over a period of 9 years. One important observation, however, is the statistically significantly lower MRC measured in the lower jaw, compared to the upper jaw in the group treated with CM. Interestingly, the increase of the KTT was similar in both groups; KTW showed only a slight difference favoring the CTG.

Most of the available literature compares different surgical techniques or reports on a single surgical technique alone and with one type of grafting material. There are only a few randomized, controlled clinical studies comparing the same surgical approach for the coverage of multiple gingival recessions using different grafting materials [15, 26–28].

The present study included multiple bilateral recessions both in the maxilla and mandible, also involving recession coverage at molars, which in turn may increase the risk of surgical difficulties and failures. Treatment of molars likely influenced the overall results because of the anatomical considerations: wide mesio-distal cervical contour, difficulties to access. Although recessions in the lateral zone may be of concern for patients with high lip lines or root hypersensitivity, they are still considered to be a major challenge for clinicians.

A recent systematic review has attempted to answer the question whether any 3D matrix biomaterial used for root coverage of localized class 1 defects may provide equivalent outcomes with CTG [29]. The results have shown that in terms of relative root coverage, no statistically significant differences were found among autogenous grafts, allografts, and xenogeneic materials. In terms of keratinized tissue width, on 2 mm recessions, CTG showed superiority above other biomaterials, but on 3 mm recessions, the results were the same. Interestingly, the percentage of recessions with CRC showed comparable results for all biomaterials.

McGuire et al. investigated the short- (up to 6 months) and the long-term outcomes (after 5 years) obtained with CM or CTG in conjunction with coronally advanced flap [30, 31] in single recessions. At 6 months, there were no statistically significant differences in terms of MRC (i.e., 97.5% for CTG and 89.5% for CM, respectively), while at 5 years the same values measured 95.5% for CTG and 77.6% for CM, respectively.

The results of the present long-term study are also in line with recent findings by Tonetti et al. [28], who have reported the 36-month follow-up of a trial comparing the adjunctive use of CM or CTG to CAF for the coverage of MAGR. At 3 years, the root coverage measured 1.5 ± 1.5 mm for CMX and 2.0 ± 1.0 mm for CTG (difference of 0.32 mm, 95% CI from − 0.02 to 0.65 mm) while the upper limit of the confidence interval was over the non-inferiority margin of 0.25 mm. Furthermore, no differences in the stability of root coverage were observed between groups over a period of 36 months. Taken together, the results suggested that CM was non-inferior to CTG for coverage of MAGR.

However, at present, to the best of our knowledge, there is no other long-term investigation which reports on data after a period of > than 5 years. In the present study, at 9 years, CRC was obtained in 2 out of the 16 patients. Interestingly, in both cases, the defects were located in the maxilla (i.e., one was treated with CM and the other one with CTG). Furthermore, in the lower jaw, only 4 out of the 42 sites showed CRC while the corresponding values for the upper jaw were 28 out of 72 sites. MRC amounted 23% in the test and 40% in the control group, respectively. The deterioration in terms of MRC and CRC measured in both the test and control group at 9 years as compared to the 12-month findings, may, on one hand, be explained by the increase in plaque levels or traumatic tooth brushing. On the other hand, it seems that the clinical outcomes are less stable in the mandibular area, compared to maxillary sites. This observation is in agreement with recent findings indicating that in cases of multiple mandibular adjacent gingival recessions, the treatment is frequently even more challenging due to difficulties related to the preparation of a tension-free flap or tunnel, stabilization of the graft and complete, and tension-free coverage of the graft and of the exposed root surfaces [32]. However, further research is needed in order to shed more light on the factors responsible for the differences in the outcomes between maxillary and mandibular recessions.

## Conclusion

Within their limits, the present results indicate that (a) the results obtained in MAGR using MCAT in conjunction with either CM or CTG are likely to deteriorate over the course of 9 years, and (b) the outcomes obtained in maxillary areas seem to be more stable compared to the mandibular ones.
